# Innovative pathological and therapeutic approaches for poorly cohesive gastric cancer

**DOI:** 10.3389/fonc.2026.1842715

**Published:** 2026-08-25

**Authors:** Margherita Muratore, Francesco Giulio Sullo, Alessandro Bittoni, Lina Cardisciani, Martina Valgiusti, Giulia Bartolini, Luca Esposito, Chiara Molinari, Richard Camara, Alessandro Passardi

**Affiliations:** 1 Department of Medical Oncology, Istituto di Ricovero e Cura a Carattere Scientifico (IRCCS) Istituto Romagnolo per Lo Studio Dei Tumori (IRST) “Dino Amadori”, Meldola, Italy; 2 Pathology Unit, Santa Maria delle Croci Hospital, Ravenna, Italy; 3 Biosciences Laboratory, Istituto di Ricovero e Cura a Carattere Scientifico (IRCCS) Istituto Romagnolo Per Lo Studio Dei Tumori (IRST) “Dino Amadori”, Meldola, FC, Italy

**Keywords:** claudin 18.2 targeted therapies, digital pathology, FGFR2 targeted therapies, HER2 targeted therapies, immunotherapy, poorly cohesive gastric cancer, predictive markers, signet ring cell carcinoma

## Abstract

Poorly cohesive gastric cancer (PCGC) represents a biologically distinct subtype of gastric cancer, characterized by diffuse growth, marked intratumoral heterogeneity, and a consistently worse prognosis compared with other histological subtypes. Despite advances in pathological classification and molecular profiling, treatment strategies remain largely independent of histological subtype, and no specific therapeutic approaches have been established for PCGC. In this review, we summarize current evidence on the biological features, diagnostic challenges, and emerging therapeutic strategies, with a focus on novel targeted agents and innovative treatment platforms. Recent years have witnessed the development of therapies directed against specific molecular targets, including HER2, PD-L1, CLDN18.2, FGFR2b, and TROP2, as well as the introduction of antibody–drug conjugates and bispecific antibodies, progressively expanding the therapeutic landscape of gastric cancer. However, their clinical impact in poorly cohesive tumors remains to be fully defined. In parallel, advances in digital pathology, artificial intelligence, and multi-omics approaches are providing new opportunities to improve diagnostic reproducibility, refine prognostic stratification, and support personalized treatment strategies by integrating histomorphological and molecular tumor features. Overall, the convergence of novel therapeutic strategies and advanced diagnostic technologies may pave the way toward a more precise and biologically informed management of PCGC, although further validation and integration into clinical practice are required.

## 1 Introduction

Poorly cohesive gastric cancer (PCGC) represents one of the most challenging histological subtypes of gastric cancer because of its aggressive biological behavior and poor overall prognosis. According to the WHO classification and the Verona consensus, PCGC includes signet-ring cell carcinoma and poorly cohesive carcinoma not otherwise specified (NOS), characterized by discohesive tumor cells infiltrating the gastric wall without gland formation (1, 2).

This histological group is characterized by diffuse infiltration of the gastric wall, marked intratumoral heterogeneity, and limited formation of glandular structures, features that contribute to delayed diagnosis and unfavorable clinical outcomes compared with intestinal-type gastric cancers (3).

Moreover, the frequently subtle and heterogeneous pathological features of poorly cohesive tumors complicate early detection, accurate diagnosis, and reliable patient stratification (2). Until recently, treatment strategies for advanced disease relied largely on systemic chemotherapy, which has historically provided limited survival benefit for this subgroup of patients (4). While treatment strategies are primarily driven by molecular biomarkers, the distinct biological and clinical features of poorly cohesive gastric cancer may still have relevant implications for disease behavior and treatment outcomes.

In recent years, however, the growing understanding of the molecular landscape of gastric cancer has led to the identification of several actionable biomarkers that are progressively reshaping the therapeutic approach to this disease (1, 5). Among the most clinically relevant targets are HER2 amplification (present in approximately 15–20% of gastric cancers), PD-L1 expression (30–60%), Claudin 18.2 (35–40%), fibroblast growth factor receptor 2 (FGFR2) alterations—including FGFR2 amplification (5–6%) and FGFR2b overexpression (up to 30–40%)—and microsatellite instability (MSI-high, approximately 9–10%) (6–10).

Each of these biomarkers is associated with specific targeted therapies or immunotherapeutic approaches that support a more personalized treatment strategy. Because the accurate detection and interpretation of these biomarkers directly influence therapeutic decision-making, the role of the pathologist has become increasingly central in the management of gastric cancer. This review aims to provide an updated overview of the main predictive biomarkers and emerging therapeutic targets in poorly cohesive gastric cancer, highlighting their biological significance, diagnostic assessment, and clinical implications.

A major challenge in the management of poorly cohesive gastric cancer (PCGC) is that most available evidence supporting biomarker-driven therapies derives from studies conducted in unselected gastric adenocarcinoma populations, with limited prospective stratification according to histological subtype. Consequently, the applicability of current targeted and immunotherapeutic strategies to PCGC remains incompletely defined. In this context, the aim of this review is not only to summarize the current evidence regarding predictive biomarkers and emerging therapeutic targets, but also to critically discuss their relevance in PCGC, highlight histology-specific evidence gaps, and explore how advances in pathological classification, digital pathology, artificial intelligence, and multi-omics approaches may contribute to a more precise characterization and management of this challenging disease.

## 2 Pathological and histological classification of poorly cohesive gastric carcinoma

Over the years, different classification systems have been used for gastric carcinoma. The Laurén classification has been one of the most widely used classification systems for gastric cancer worldwide since its publication in 1965. Laurén distinguished for the first time intestinal-type adenocarcinomas and diffuse-type carcinomas (11). Intestinal-type adenocarcinomas are the most common histotype and generally show a more favorable prognosis than other types (12). Diffuse-type carcinomas, in contrast, occur in younger patients and have a more aggressive clinical behavior (13). In several studies, this classification has demonstrated—even after decades—to correlate not only with prognosis but also with molecular features and response to therapy (14).

In 2019, the classification of gastric tumors proposed by the World Health Organization (WHO) in its 5th edition integrated classic morphological aspects with increasingly relevant molecular features (15). Unlike the Laurén classification, which simply distinguishes intestinal and diffuse subtypes, the WHO classification identifies several different subtypes of gastric adenocarcinoma, the main subtypes being tubular, papillary and mucinous adenocarcinoma, as well as poorly cohesive carcinoma (including signet-ring cell carcinoma). The classification also includes other rare histotypes that display specific histological and clinical characteristics (15).

Poorly cohesive gastric carcinomas (PCGC) represent a numerically significant (20–54%) and heterogeneous group of gastric carcinomas, including all tumors in which cells lack cohesion and do not form glands. Signet-ring cell (SRC) carcinoma is the main variant, but the group also includes tumors composed of non-mucin-secreting cells with a diffuse growth pattern. Since the 2019 WHO edition, some issues have persisted in PCGC classification, especially concerning the marked morphological heterogeneity observed within individual lesions, reflecting the presence of multiple subclonal populations. For this reason, the new consensus from the World Gastric Carcinoma Group emphasized the distinction between SRC and other forms of PCGC, including the so-called NOS (not otherwise specified) subtype (1, 2).

The concept of “poorly cohesive NOS” implies the presence of an isolated tumor cell population with scant cytoplasm and scant intracellular mucin, with a central nucleus and widespread atypia. This definition morphologically distinguishes NOS cells from SRC, which is characterized by intracellular mucin and an eccentric nucleus, effectively dividing PCGC into two main phenotypes. A challenge posed by this new classification is represented by the paradigm shift in the differential diagnosis between poorly differentiated adenocarcinomas with solid architecture and no recognizable tubules and PCGC of the NOS subtype (16). This distinction is important, since increasing evidence suggests a better prognosis in cases with a prevalent adenocarcinoma component, as well as different poorly cohesive phenotypes in mixed carcinomas (17, 18).

### 2.1 Tissue sampling and pre-analytical factors in the era of predictive markers

The last decade has witnessed the rise and consolidation of targeted therapies for many human solid malignancies, including gastric cancer. This revolution has implied a central role for the pathologist due to the necessity of evaluating tissue markers predictive of response to targeted therapy. In particular, immunohistochemistry for HER2, PD-L1, Claudin 18.2 (CLDN18.2), and the four main proteins of the mismatch repair system (MMR)—MLH1, PMS2, MSH2 and MSH6—is required either at the time of diagnosis or upon request by the oncologist (19).

This means that at least 20 µm of tissue must be cut from the paraffin block, to which additional 10-µm sections for molecular biology must be added when required.

The need for sufficient material for these techniques, together with the intrinsic tumor heterogeneity of PCGC, has increased the necessity for more rational and standardized biopsy sampling by the endoscopist or gastroenterologist. Early studies demonstrated that two biopsies of the tumor lesion significantly improved diagnostic accuracy (20), but the most recent guidelines suggest performing at least six biopsies, not only for diagnostic purposes but also to preserve sufficient material for immunohistochemistry and molecular biology (19).

Once the quantity of material has been secured, another important—often underestimated—aspect of routine pathology practice is represented by the many pre-analytical issues that can invalidate diagnosis and/or immunohistochemistry. These practices have been demonstrated to be particularly important for HER2 and MMR proteins and are endorsed by major institutions (21–23).

For other markers, such as PD-L1 and CLDN18.2, it is strongly recommended not to use old material (e.g., pre-cut blank sections or tissue blocks older than two years), since antigen preservation may not be guaranteed (24, 25).

## 3 Predictive biomarkers and therapeutic targets in poorly cohesive gastric carcinoma

### 3.1 HER2: its role in the treatment of poorly cohesive gastric carcinoma

Human epidermal growth factor receptor 2 (HER2) represents a critical therapeutic target in advanced gastric cancer (AGC). Since the publication of the ToGA trial, trastuzumab in combination with chemotherapy has been established as the standard of care for patients with HER2-positive AGC for more than a decade (26).

HER2 positivity is observed in approximately 15% of gastric and gastroesophageal junction cancers. Several studies investigating clinicopathological correlations have demonstrated a strong association between HER2 amplification and both tumor location and histological subtype. In particular, Tanner et al. reported HER2/neu amplification in 16 of 131 gastric adenocarcinomas (12.2%) and in 24 of 100 gastroesophageal adenocarcinomas (24.0%). Notably, HER2/neu amplification was significantly more frequent in intestinal-type gastric cancers (21.5%) compared with diffuse (2%) or mixed/anaplastic subtypes (p = 0.0051) (27). Consistent results were observed in a Korean study of 182 patients who underwent gastrectomy, in which intestinal-type tumors exhibited higher rates of HER2 amplification than diffuse-type tumors (p < 0.05) (28). These findings were further confirmed in the large cohort of patients screened for the ToGA trial, where HER2 positivity differed significantly according to histological subtype (intestinal 34%, diffuse 6%, mixed 20%) (29).

As a consequence, only a small proportion of patients enrolled in the ToGA trial (approximately 9%) had diffuse-type tumors. Interestingly, the overall survival benefit associated with trastuzumab was more pronounced in patients with intestinal-type disease (HR 0.69, 95% CI 0.54–0.88) than in those with diffuse-type tumors (HR 1.07, 95% CI 0.56–2.05).

The biological mechanisms underlying the preferential overexpression of HER2 in intestinal-type gastric carcinoma remain incompletely understood but likely reflect distinct molecular pathways across histological subtypes. For instance, HER2 gene amplification in gastric cancer is known to be inversely associated with E-cadherin mutations, which represent a hallmark molecular alteration of diffuse-type disease (30).

In recent years, the development of novel anti-HER2 agents and innovative therapeutic combinations has yielded encouraging results, offering promising new treatment opportunities for patients with HER2-positive gastric cancer. In particular, based on the results of the KEYNOTE-811 trial, patients with HER2-positive and PD-L1–positive (CPS ≥1) AGC may now benefit from the addition of the immune checkpoint inhibitor pembrolizumab to trastuzumab and chemotherapy in the first-line setting (31).

Trastuzumab deruxtecan (T-DXd) is an antibody–drug conjugate composed of a humanized anti-HER2 monoclonal antibody linked to a topoisomerase I inhibitor payload. Following the promising results observed in the phase II DESTINY-Gastric01 and DESTINY-Gastric02 trials (32, 33), the phase III DESTINY-Gastric04 trial evaluated the efficacy and safety of trastuzumab deruxtecan compared with ramucirumab plus paclitaxel as second-line therapy in patients with HER2-positive advanced gastric cancer previously treated with trastuzumab (34).

Among the most promising investigational anti-HER2 therapies, zanidatamab represents one of the most interesting agents. Zanidatamab is a bispecific monoclonal antibody that binds two distinct domains of the HER2 receptor. In a recent phase II trial in first-line treatment of HER2-positive metastatic gastric cancer, zanidatamab combined with chemotherapy demonstrated a confirmed ORR of 76%, which reached 84% among centrally confirmed HER2-positive patients, while the median duration of response was 18.7 months (35).

More recently, results of the primary analysis of the phase III HERIZON-GEA-01 trial have been presented at ASCO GI 2026 (36).

Overall, HER2 positivity appears to be uncommon in poorly cohesive gastric carcinoma, reflecting its preferential association with intestinal-type gastric cancer. HER2 remains a well-established predictive biomarker for anti-HER2 therapies in gastric cancer and currently represents a key driver of treatment selection irrespective of histological subtype. However, the specific impact of poorly cohesive histology on treatment outcomes remains insufficiently characterized, as no prospective studies have been specifically designed for this patient population.

Summary of studies investigating anti-HER2 therapies in **Table 1**.

**Table 1:** Clinical studies evaluating HER2-targeted therapies in gastric or gastroesophageal junction cancer, including available histological subgroup data.

Study	Design	Setting	Treatment	Primary end point	Secondary end point	Histology subtype
ToGA Trial (29)	Phase III	I line	FP + trastuzumab vs FP	OS 13.8. vs 11.1 HR 0.74	PFS 6.7 vs 5.6 HR 0.71 TTP 7.1 vs 5.6 HR 0.70 ORR 47% vs 35%	Intestinal: 77% HR 0.69 (0.54 – 0.88) Diffuse: 9% HR 1.07(0.56 – 2.05) Mixed: 14% HR 0.86 (0.51 – 1.46)
Keynote 811 (31)	Phase III	I line	fluoropyrimidine and platinum-based therapy + trastuzumab + pembrolizumab vs fluoropyrimidine and platinum-based therapy + trastuzumab	OS 20 vs 16.8 HR 0.8 PFS 10 vs 8.1 HR 0.8	ORR 74% vs 51.7% DCR 96% vs 89% DoR 11.3 vs 9.5	Intestinal: 57% HR 0.73 (0.47 – 1.07) Diffuse: 19% HR 0.71 (0.47 – 1.07) Indeterminate: 24% HR 0.81 (0.59 – 1.12)
Destiny Gastric 01 (32)	Phase II	III line	T-DXd vs irinotecan or paclitaxel	ORR 51% vs 14%	OS 12.5 vs 8.4 HR 0.59 PFS 5.6 vs 3.5 HR 0.47 DoR 11.3 vs 3.9	Intestinal: 71% Diffuse: 22% Other: 6%
Destiny Gastric 02 (33)	Phase II	II line	T-DXd	ORR 42%	DoR 8.1 PFS 5.6 OS 12.1	Intestina: 24.1% Diffuse: 1.3% Mixed: 1.3% Unknown: 72.2%
Destiny Gastric 04 (34)	Phase III	II line	T-DXd vs paclitaxel and ramucirumab	OS 14.7 vs 11.4 HR 0.70 PFS 6.7 vs 5.6 HR 0.74	ORR 44.3% vs 29.1%	No data
Herizon-Gea-01 (36)	Phase III	I line	fluoropyrimidine and platinum-based therapy + trastuzumab vs fluoropyrimidine and platinum-based therapy + zanidatamab vs fluoropyrimidine and platinum-based therapy + zanidatamab + tislelizumab	OS 19.2 vs 24.4 vs 24.6 PFS 8.1 vs 12.4 vs 12. 4	ORR 65.7% vs 69.6% vs 70.7% DoR 8.3 vs 14.3 vs 20.7	No data

FP, fluorouracil and cisplatin; T-DXd, trastuzumab deruxtecan; OS, overall survival; PFS, progression free survival; ORR, objective response rate; TTP, time to progression; DoR, duration of response; HR, hazard ratio; DCR, disease control rate.

### 3.2 PD-L1: its role in the treatment of poorly cohesive gastric carcinoma

Programmed cell death protein 1 (PD-1) is a regulator of T cell function. Upon binding with programmed cell death-ligands 1 and 2 (PD-L1 and PD-L2), expressed on cancer cells, PD-1 activation inhibits T cell receptor signaling and modulates pathways that ultimately allow tumors to escape immune surveillance (37). Several immunotherapeutic agents, known as immune checkpoint inhibitors (ICIs), have been developed to inhibit PD-1 signaling targeting either PD-1 or PD-L1. They have proven effective, alone or in combination with chemotherapy or other agents including ICIs against different targets (e.g. anti-CTLA-4), in several cancer types such as melanoma, non-small cell lung cancer, hepatocellular carcinoma, biliary tract cancer (38–41).

Given the abovementioned mechanism of action, PD-L1 expression has been proposed as a predictive factor for ICI activity. With the aim to obtain a quantitative evaluation of PD-L1 expression, several methods for immunohistochemical analysis have been developed. Specifically in gastric and gastroesophageal junction (GEJ) carcinoma, two different definitions of PD-L1 expression have been used in clinical trials, i.e. combined positive score (CPS) and tumor area positivity (TAP) score, both evaluated by immunohistochemistry on FFPE tissue. For ease of clinical interpretation, the definitions of CPS and TAP score are summarized below.


$$CPS=\frac{\text{PD}‐\text{L}1‐\text{positive cells }(\text{tumor cells}+\text{lymphocytes}+\text{macrophages})}{\text{total number of viable tumor cells}}\times 100TAP=\frac{area occupied by PD‐L1‐\text{positive cells}}{\text{total tumor area}}\times 100$$

However, it is worth noting that, at least in patients treated with the anti-PD-1 tislelizumab, a good concordance has been shown for PD-L1 determination with either TAP or CPS score. This consistency has been observed with different PD-L1 cutoffs and has been translated into consistent clinical outcomes in cohorts including patients from RATIONALE-305 (42, 43).

In gastric carcinoma, PD-L1 positivity has been reported as a poor prognostic factor along with diffuse histology (44). Moreover, the combination with CD8+ tumor infiltrating lymphocytes represents an even stronger prognostic factor (44). The poor prognostic impact of PD-L1 positivity has been confirmed by a meta-analysis encompassing 15 studies (45).

Diffuse-type gastric cancer (DGC) is typically associated with lower sensitivity to chemotherapy and ICIs (46, 47). Furthermore, signet ring cell carcinomas show lower response rates and decreased PFS and OS compared with non-signet ring cell tumors (48).

Several tissue features of DGC have been associated with a possible reduced effect of immunotherapy. Interestingly, the poorly cohesive components are located in the deeper layers of diffuse gastric carcinoma, and PD-L1 is also specifically expressed in the deep layer, reflecting deep layer-specific immunosuppression (49).

Tumor microenvironment is a key determinant of the cancer-immune interface. In diffuse subtype, a more isolated immune infiltration close to poorly cohesive cells has been identified, along with higher abundance of cancer-associated fibroblasts and lower presence of cytotoxic T cells compared with intestinal subtype (48, 50). These characteristics contribute to the “cold tumor” phenotype observed in diffuse gastric cancer.

With respect to available evidence, there are no trials that specifically evaluated ICIs in poorly cohesive gastric carcinoma. Data therefore derive from subgroup analyses of trials including both intestinal and diffuse histologies (48).

CheckMate-649 demonstrated that nivolumab plus chemotherapy significantly improved OS and PFS compared with chemotherapy alone in patients with PD-L1 CPS ≥5 (51). Long-term follow-up confirmed these results with persistent survival benefit (52).

The phase III KEYNOTE-859 trial demonstrated that the addition of pembrolizumab to chemotherapy significantly improved overall survival (OS) across all analyzed populations (53).

Subgroup analyses from KEYNOTE-062 suggested a potential benefit of pembrolizumab, particularly in patients with diffuse-type gastric cancer (54). However, these findings should be interpreted with caution, as they derive from exploratory analyses that were not specifically powered for histological subtypes.

In HER2-positive advanced gastric cancer, the KEYNOTE-811 trial evaluated the combination of pembrolizumab, trastuzumab, and chemotherapy, showing improved clinical outcomes compared with standard therapy (31, 55). Similarly, the RATIONALE-305 study demonstrated a survival benefit with the addition of tislelizumab to chemotherapy in patients with PD-L1–positive tumors (42).

In the perioperative setting, the MATTERHORN trial showed that the addition of durvalumab to FLOT chemotherapy significantly improved event-free survival (EFS) compared with chemotherapy alone (56). Conversely, the KEYNOTE-585 trial failed to demonstrate a significant improvement in EFS with perioperative pembrolizumab (57).

Importantly, none of the major phase III trials evaluating immune checkpoint inhibitors in gastric cancer were specifically designed to assess efficacy in poorly cohesive gastric carcinoma. Therefore, available evidence in this histological subtype is limited to exploratory subgroup analyses, which are often underpowered and inconsistently reported. As a result, the clinical benefit of immunotherapy in poorly cohesive and signet-ring cell gastric cancers remains uncertain and represents an important unmet need for future prospective studies.

Overall, PD-L1 remains an established predictive biomarker for immune checkpoint inhibitors in advanced gastric cancer, irrespective of histological subtype. However, poorly cohesive and diffuse-type gastric cancers are characterized by distinctive tumor microenvironment features, including reduced cytotoxic immune infiltration and increased stromal components, which may influence treatment sensitivity. While current evidence supports the use of immunotherapy in biomarker-selected patients, the specific impact of poorly cohesive histology on immunotherapy outcomes remains incompletely characterized, as no prospective studies have been specifically designed for this patient population.

Summary of studies investigating immunotherapy in **Table 2**.

**Table 2:** Clinical studies evaluating immune checkpoint inhibitors in gastric or gastroesophageal junction cancer, including available histological subgroup data.

Study	Design	Setting	Treatment	Primary end point	Secondary end point	Histology subtype
Check Mate 649 (51)	Phase III	I line	folfox + nivolumab vs folfox	OS in pz with CPS ≥ 5 14.4 vs 11.1 HR 0.71 Pfs in pz with CPS ≥ 5 7.7 vs 6 HR 0.68	ORR 59.7% vs 44.4% (CPS ≥ 5) DoR 9.6 vs 7 (CPS ≥ 5)	Intestinal: 36% Diffuse: 29% Mixed: 8% Unknown: 27%
Keynote 859 (53)	Phase III	I line	capox or FP + pembrolizumab vs capox or FP	OS ITT 12.9 vs 11.5 HR 0.78 CPS ≥ 1–13 vs 11.4 HR 0.74 CPS ≥ 10 15.7 vs 11.8 HR 0.63	PFS (ITT population) 6.9 vs 5.6 HR 0.76 ORR 52% vs 42% DoR 8 vs 6.9	Intestinal: 36% HR 0.81 (0.67 – 0.98) Diffuse 40% HR 0.76 (0.64 – 0.90) Indeterminate: 24% HR 0.69 (0.55 – 0.87)
Rationale 305 (42)	Phase III	I line	capox or FP + tislelizumab vs capox or FP	OS ITT 15 vs 12.9 HR 0.80 TAP ≥ 5 17.2 vs 12.6 HR 0.74	PFS 7.2 vs 6.3 HR 0.74 ORR 54.6% vs 5.6% DoR 7.2 vs 5.6	No data
Matterhorn (56)	Phase III	Peri-operative	FLOT + durvalumab vs FLOT	EFS NR vs 32.8 HR 0.71	pCR 19 vs 7 OS NR vs 47.2 HR 0.78	Intestinal: 51.7% HR 0.66 (0.48 – 0.89) Diffuse: 27.4% HR 0.93 (0.66 – 1.32) Mixed/other: 20.9% HR 0.56 (0.36 – 0.86)
Keynote 585 (57)	Phase III	Peri-operative	capox or FP + pembrolizumab vs capox or FP and FLOT + pembrolizumab vs FLOT	pCR main cohort 13.4% vs 2% Main cohort + FLOT 14.2% vs 2.8% OS 71.8 vs 55.7 HR 0.86 EFS 44.4 vs 25.5 HR 0.81	R0 81.2% vs 78.1%	Diffuse: 41% Intestinal: 48% Mixed/Unknown:11%

FP, fluorouracil and cisplatin; OS, overall survival; PFS, progression free survival; ORR, objective response rate; DoR, duration of response; HR, hazard ratio; CPS, combined positive score; ITT, intention to treat; TAP, tumor area positivity; EFS, event free survival; NR, not reached; pCR, patological complete response; R0, resection with no residual tumor.

### 3.3 Claudin 18.2: its role in the treatment of poorly cohesive gastric carcinoma

Claudin 18 isoform 2 (CLDN18.2) is a tight junction protein that is strictly involved in signal transduction, regulation of tissue permeability and paracellular communication (58). CLDN18.2 is physiologically expressed in stomach and gastro-esophageal mucosa and its upregulation is related to loss of cellular polarity and activation of proliferative signals in malignant transformation; these events lead to disruption of cellular bonds and expose this protein on tumor cell surface, making it an attractive target for therapeutic agents (59).

CLDN18.2 is overexpressed in up to 40% of gastric and gastroesophageal junction (G/GEJ) cancers and appears to be slightly more prevalent in younger and female patients (60, 61). Although it is involved in tumor-associated alterations of epithelial polarity, CLDN18.2 overexpression has not been consistently associated with prognostic impact in gastric cancer, nor has it demonstrated a predictive role for response to immunotherapy in the metastatic setting (62–64).

Notably, some studies have suggested a trend toward worse survival in patients with diffuse-type gastric cancer exhibiting high CLDN18.2 expression; however, this observation did not reach statistical significance and should be interpreted with caution (62).

Despite the lack of clear prognostic significance, CLDN18.2-positive advanced gastric cancer appears to be associated with a higher incidence of peritoneal metastases, and peritoneal lesions show greater concordance of CLDN18.2 expression with the primary tumor compared with other metastatic sites (65).

Zolbetuximab is an anti-CLDN18.2 monoclonal antibody approved in combination with chemotherapy in patients with HER2-negative, CLDN18.2-positive advanced GC/GEJC (58).

Several studies questioned the appropriate cut-off to define when CLDN18.2 is overexpressed.

The FAST trial is a phase II study that compared zolbetuximab plus chemotherapy (EOX) versus chemotherapy alone as a first-line treatment for advanced CLDN18.2-positive GC/GEJC. CLDN18.2 positivity was set as moderate-to-strong expression of CLDN18.2 in ≥40% tumor cells. This trial showed improved PFS in the zolbetuximab plus EOX arm (7.5 vs. 5.3 months) with a median OS of 13.0 months compared to 8.3 months in the chemotherapy-alone group (66). Subgroup analysis showed that patients with CLDN18.2 expression in ≥70% tumor cells were the only group with significant improvements in PFS and OS.

These results helped the next studies to move forward cut-off positivity for CLDN18.2 overexpression to ≥75% cells.

The GLOW trial is a phase III study that enrolled 507 patients with HER2-negative, locally advanced or metastatic GC/GEJC, randomized to receive either zolbetuximab plus CAPOX or CAPOX plus placebo. Zolbetuximab addition resulted in improving PFS (8.21 vs. 6.80 months) and OS (14.39 vs. 12.16 months) in the first-line setting (67).

The SPOTLIGHT trial is a phase III study that investigated zolbetuximab efficacy in first-line setting when added to FOLFOX. Zolbetuximab significantly improved PFS (10.6 vs. 8.6 months) and OS (18.2 months vs. 15.5 months) (68).

Frequency of CLDN18.2 overexpression and efficacy of CLDN18.2-target agents in PCC remain indefinite due to lack of specific clinical trials in this subpopulation of GC.

Neither SPOTLIGHT nor GLOW study planned *ad-hoc* analysis for histological subgroup; nonetheless both studies reported a non-significant improvement in OS by addition of zolbetuximab to chemotherapy in diffuse-type GC (67, 68).

Recently, a single-center study retrospectively analyzed 178 chemo-naïve resected stage II–III PCC cases and investigated CLDN18.2 positive percentage (≥75% positive cells). CLDN18.2 overexpression was found in approximately one-third of PCC cases and showed no association with sex or age (69).

Absence of correlation between CLDN18.2 overexpression and metastatic PCC was also underlined by an Italian study (70).

Further studies are needed to clarify the biological and therapeutic relevance of CLDN18.2 in this subpopulation characterized by poor clinical outcomes and limited actionable targets.

CLDN18.2 expression appears to be relatively frequent in poorly cohesive gastric carcinoma, being detected in approximately one-third of reported cases. Although its prognostic significance remains uncertain, CLDN18.2 is an established predictive biomarker for zolbetuximab-based therapies in gastric cancer. However, the clinical relevance and predictive value of CLDN18.2 specifically in PCGC remain incompletely defined, as prospective studies evaluating CLDN18.2-targeted therapies according to histological subtype are currently lacking.

Summary of studies investigating anti CLDN18.2 therapies in **Table 3**.

**Table 3:** Clinical studies evaluating CLDN18.2-targeted therapies in gastric or gastroesophageal junction cancer, including available histological subgroup data.

Study	Design	Setting	Treatment	Primary endpoint	Secondary endpoint	Histology subtype
FAST (66)	Phase II	I line	EOX + zolbetuximab vs EOX	PFS 7.5 vs 5.3 HR 0.44	OS 13 vs 8.3 HR 0.55 ORR 39% vs 25%	Diffuse: 45.5% Intestinal: 33.8% Mixed: 13% Unknown: 7.8%
GLOW (67)	Phase III	I line	capox + zolbetuximab vs capox	PFS 8.21 vs 6.8 HR 0.687 OS 14.39 vs 12.6 HR 0.771	ORR 42.5% vs 40.3% DoR 8.5 vs 6.6	Diffuse: 37% HR 0.62 (0.411 – 0.936) Intestinal: 15.2% HR 0.675 (0.375 – 1.217) Mixed/other: 35.8% HR 0.824 (0.499 – 1.358)
SPOTLIGHT (68)	Phase III	I line	folfox + zolbetuximab vs folfox	PFS 10.61 vs 8.67 HR 0.75 OS 18.23 vs 15.54 HR 0.75	ORR 61.8% vs 50.3% DoR 8.5 vs 6.8	Diffuse: 29% HR 0.76 (0.51 – 1.13) Intestinal: 25% HR 0.58 (0.38 – 0.89) Mixed/other: 46% HR 0.93 (0.60 – 1.43)
TRANSTAR 102 – gastric cohort (71)	Phase I/II	I line	TST001 + capox vs TST001 + nivolumab + capox	Safety G≥3 AEs: 54.5% vs 57.1%	ORR 43.2% vs 55.7% (67.6% high CLDN 18.2)	No data
ILUSTRO Cohort 4 (72)	Phase II	I line	folfox + zolbertuximab + nivolumab	ORR 68.1% (in high CLDN 18.2	PFS 23.6 in PDL1 ≥ 1 12.1 PDL1< 1	Diffuse: 57.1% Intestinal: 33.3% Other: 9.5%
LUCERNA	Phase III	I line	Folfox + zolbetuximab + pembrolizumab	OS, PFS ongoing trial	ORR, DoR, DCR ongoing trial	No data

OS, overall survival; PFS, progression free survival; ORR, objective response rate; DoR, duration of response; HR, hazard ratio; DCR, disease control rate.

### 3.4 FGFR2: its role in the treatment of poorly cohesive gastric carcinoma

Fibroblast growth factor receptors (FGFRs) are a family of transmembrane receptor tyrosine kinases that regulate key cellular processes, including proliferation, survival, migration, and differentiation. Upon ligand binding, FGFRs activate multiple downstream signaling cascades, most notably the RAS/MAPK, PI3K/AKT, JAK/STAT, and PLCγ pathways, thereby exerting a critical role in tissue homeostasis and oncogenesis (73, 74).

In cancer, aberrant FGFR signaling is frequently driven by genetic alterations such as activating mutations, gene amplification, or receptor overexpression. Among FGFR family members, FGFR2 dysregulation has emerged as a relevant oncogenic driver in gastric cancer. Large-scale genomic studies, including analyses from The Cancer Genome Atlas (TCGA), have reported FGFR2 gene amplification in approximately 4–10% of gastric adenocarcinomas, with a marked enrichment in diffuse-type and poorly cohesive histologies (75).

In contrast, FGFR2 amplification is rarely observed in intestinal-type tumors and is often mutually exclusive with other actionable molecular alterations, such as HER2 amplification or microsatellite instability, suggesting that FGFR2 defines a distinct molecular subtype of gastric cancer (76–78).

FGFR2 gene amplification results in receptor overexpression and constitutive activation of oncogenic signaling pathways, thereby promoting tumor initiation and progression. Clinically, FGFR2-amplified gastric cancers are more frequently characterized by high histological grade, advanced tumor stage, lymph node involvement, and an increased incidence of distant and peritoneal metastases. Several retrospective studies have consistently shown an association between FGFR2 amplification and poor clinical outcomes, including reduced overall survival, particularly in patients with diffuse-type gastric cancer (78–80).

Moreover, FGFR2 amplification has been linked to early disease recurrence and resistance to platinum-based chemotherapy regimens, highlighting its role as both a prognostic and potentially predictive biomarker (81, 82).

Beyond gene amplification, FGFR2 protein overexpression, as assessed by immunohistochemistry (IHC), has been reported in approximately 31–61% of gastric cancer cases. FGFR2 overexpression correlates with aggressive clinicopathological features, including increased depth of tumor invasion, lymph node and distant metastases, and inferior overall survival, although it has not yet been established as a reliable predictor of chemotherapy response (79, 83).

FGFR2 signaling is further modulated through alternative splicing, which produces isoforms with distinct ligand-binding properties. The epithelial isoform FGFR2b preferentially binds FGFs 1, 3, 7, 10, and 22, whereas the mesenchymal isoform FGFR2c interacts with a wider spectrum of FGFs. In gastric cancer, FGFR2b represents the predominant overexpressed isoform, showing a strong correlation with FGFR2 gene amplification and poorer clinical outcomes (84, 85).

Over the past decade, multiple approaches have been developed to target FGFR2 in gastric cancer, including small-molecule tyrosine kinase inhibitors (TKIs) and monoclonal antibodies. TKIs such as AZD4547, futibatinib, infigratinib, and pemigatinib act by binding the intracellular tyrosine kinase domain of FGFR2, thereby preventing receptor phosphorylation and downstream signaling.

Several therapeutic strategies targeting FGFR2 have been developed, including small-molecule tyrosine kinase inhibitors (TKIs) and monoclonal antibodies.

Among TKIs, AZD4547 demonstrated significant antitumor activity in preclinical FGFR2-amplified models (86); however, these promising results were not confirmed in the phase II SHINE trial, which failed to show a significant improvement in progression-free survival compared with paclitaxel (87).

Other FGFR inhibitors have shown more encouraging signals. Futibatinib, an irreversible FGFR1–4 inhibitor, achieved tumor shrinkage in more than half of patients with FGFR2-amplified gastric cancer in phase II studies (88, 89). Similarly, infigratinib demonstrated objective responses in patients with FGFR2-amplified gastric or gastroesophageal junction adenocarcinoma (90), while pemigatinib has also shown clinical activity in FGFR2-altered gastric cancer (91).

In parallel, monoclonal antibodies targeting FGFR2b have emerged as a promising therapeutic approach. Among these, bemarituzumab has achieved the most advanced clinical development. Preclinical studies demonstrated effective inhibition of FGFR2 signaling along with enhanced antibody-dependent cellular cytotoxicity (92). Early-phase clinical evaluation confirmed antitumor activity, with partial responses observed in patients with FGFR2-amplified tumors (93).

These findings were further supported by the phase I/II FIGHT trial, which showed improved overall survival in patients treated with bemarituzumab in combination with chemotherapy (94, 95). Building on these results, the phase III FORTITUDE-101 trial subsequently evaluated bemarituzumab plus chemotherapy in patients with FGFR2b-overexpressing gastric cancer (96). Although bemarituzumab has demonstrated improved survival outcomes in both the phase I/II FIGHT trial and the phase III FORTITUDE-101 study, these benefits were primarily driven by biomarker-selected populations, and no dedicated subgroup analyses according to histological subtype have been clearly reported. As a result, the clinical relevance of FGFR2b-targeted therapy in diffuse and poorly cohesive gastric cancer remains to be fully defined, despite a compelling biological rationale.

In addition to these approaches, combination strategies are being actively explored, particularly the integration of FGFR2-targeted therapies with immune checkpoint inhibitors, supported by a potential synergistic antitumor effect (97). Moreover, next-generation therapeutic strategies—including antibody–drug conjugates and targeted protein degradation approaches—are under development, with the aim of overcoming resistance mechanisms and enhancing the durability of clinical benefit (98, 99).

Among currently emerging therapeutic targets, FGFR2 represents one of the most biologically relevant alterations in poorly cohesive gastric cancer, given its enrichment in diffuse-type histology and its association with aggressive clinicopathological features and unfavorable outcomes. While FGFR2 alterations appear to have both prognostic and therapeutic relevance, evidence supporting FGFR2-targeted therapies in PCGC remains limited to biomarker-selected studies not specifically designed according to histological subtype. Therefore, the precise clinical impact of FGFR2-directed strategies in poorly cohesive gastric cancer remains to be established.

Summary of studies investigating anti FGFR2 therapies in **Table 4**.

**Table 4:** Clinical studies evaluating FGFR2-targeted therapies in gastric or gastroesophageal junction cancer, including available histological subgroup data.

Study	Design	Setting	Treatment	Primary endpoint	Secondary endpoint	Histology subtype
FIGHT (94)	Phase II	I line	folfox + bemarituzumab vs folfox	PFS 9.5 vs 7.4 HR 0.68	OS 19.2 vs 13.5 HR 0.58 ORR 47% vs 33%	Diffuse: 36% Intestinal: 21% Mixed/other: 42%
FORTITUTE 101 (96)	Phase III	I line	folfox + bemarituzumab vs folfox	OS in FGFR2b≥ 10% 2+/3+TC 14.5 vs 13.2 HR 0.82	ORR in FGFR2b≥ 10% 2+/3+TC 45.9% vs 44.8% PFS in FGFR2b≥ 10% 2+/3+TC 8.6 vs 6.7 HR 0.71	No data
NCT04189445 (89)	Phase II	≥ II line	futibatinib	ORR 17.9%	DCR 50% PFS 2.9 OS 5.9	No data
NCT05019794 (90)	Phase II	≥ II line	infingratinib	ORR 25%	PFS 3.7 OS 7.5 DCR 83.3%	No data

OS, overall survival; PFS, progression free survival; ORR, objective response rate; HR, hazard ratio; DCR, disease control rate.

### 3.5 MSI-H: its role in the treatment of poorly cohesive gastric carcinoma

From a molecular perspective, gastric cancer (GC) has been stratified into distinct genomic subtypes through large-scale integrative analyses. The Cancer Genome Atlas (TCGA) project identified four principal categories: Epstein–Barr virus (EBV)–positive tumors, characterized by extensive DNA hypermethylation, recurrent *PIK3CA* mutations, and amplification of *JAK2*, *PD-L1*, and *PD-L2*; microsatellite instability (MSI) tumors, enriched for mutations affecting targetable oncogenic signaling pathways and generally associated with a more favorable prognosis (10); chromosomal instability (CIN) tumors; and genomically stable (GS) tumors, the latter being enriched for diffuse histology and alterations in cell adhesion–related genes.

Subsequently, complementary molecular classifications have further refined GC heterogeneity, identifying a mesenchymal-like subtype—largely overlapping with diffuse-type tumors and including the majority of poorly cohesive carcinomas—alongside MSI, TP53-active, and TP53-inactive subgroups with distinct clinical behaviors (5).

Poorly cohesive gastric carcinomas (PCC-GC) predominantly fall within the genomically stable (GS) subtype; however, a minority display MSI or EBV-positive molecular profiles, both of which are associated with increased sensitivity to immune checkpoint inhibition and therefore carry potential therapeutic implications. Consistently, Hirotsu et al. reported that MSI-H occurs only at low frequencies (around 6%) in PCC-GC, indicating that while clinically relevant, MSI does not represent a common molecular feature of this histological subtype (100).

Clinically, localized dMMR/MSI gastric adenocarcinomas are consistently associated with a favorable prognosis and appear to derive limited or no benefit from perioperative or adjuvant fluoropyrimidine-based chemotherapy, as suggested by retrospective analyses of randomized trials (5). These observations have supported the investigation of chemotherapy-sparing strategies based on immune checkpoint blockade in this population.

In this context, the phase II NEONIPIGA (NCT04006262) trial evaluated a perioperative immunotherapy approach with neoadjuvant nivolumab plus ipilimumab followed by adjuvant nivolumab in patients with resectable cT2–T4 dMMR/MSI gastric or gastroesophageal junction adenocarcinoma, reporting a pathological complete response rate of 58.6% among the 29 analyzed patients (101).

Similarly, the phase II INFINITY (NCT04817826) study assessed neoadjuvant durvalumab plus tremelimumab and demonstrated a comparable pathological complete response rate of approximately 60% (9/15 patients), further supporting the high sensitivity of localized MSI-H tumors to dual immune checkpoint inhibition (102).

Overall, MSI-H/dMMR status, while relatively rare in poorly cohesive gastric cancer, remains an important predictive biomarker, given the favorable response of MSI-H/dMMR tumors to immune checkpoint inhibitors and the improved prognosis generally observed in this molecular subgroup. However, the specific impact of poorly cohesive histology on immunotherapy outcomes remains incompletely characterized, as no prospective studies have been specifically designed for this patient population.

## 4 Future directions

### 4.1 Emerging targets and therapeutic strategies

In gastric cancer, novel therapeutic strategies based on combination approaches are emerging. The phase II ILUSTRO study evaluated the combination of mFOLFOX6, zolbetuximab, and nivolumab, showing promising activity in patients with high CLDN18.2 expression, with an objective response rate (ORR) of 68.6% and a median progression-free survival (PFS) of 14.8 months (72). Results from the ongoing phase III LUCERNA trial are eagerly awaited; this study is investigating the addition of zolbetuximab and pembrolizumab to chemotherapy in patients with metastatic gastric cancer with high CLDN18.2 expression and PD-L1 positivity (103). In parallel, the phase III trial NCT06093425 will evaluate a similar strategy using osemitamab, a next-generation, humanized IgG1 anti-CLDN18.2 monoclonal antibody, in combination with pembrolizumab and chemotherapy as first-line treatment for advanced or metastatic gastric cancer (104). Notably, this study was designed on the basis of emerging data from cohort G of the phase I/II TranStar102 study, which demonstrated a confirmed objective response rate (ORR) of 68% and a median progression-free survival (PFS) of 16.6 months (95% CI, 5.8–21.7) in patients with known CPS and high/medium CLDN18.2 expression (defined as membranous CLDN18.2 staining ≥2+ in ≥40% of tumor cells by central immunohistochemistry), with a manageable safety profile consistent with the drug class (71).

In addition to CLDN18.2-directed strategies, there are also ongoing trials evaluating the combination of immunotherapy with agents targeting alternative oncogenic pathways. In this context, the phase III FORTITUDE-102 trial is evaluating nivolumab in combination with bemarituzumab in patients with FGFR2b-overexpressing gastric cancer; results are awaited (105).

Another emerging target under investigation in gastric cancer is TROP2, a transmembrane glycoprotein highly expressed in several epithelial tumors and associated with tumor proliferation and poor prognosis (106). TROP2-targeting antibody–drug conjugates are also being investigated in metastatic gastric cancer. The phase I/II open-label MK-3475-06C/KEYMAKER-U06 study is evaluating sacituzumab tirumotecan (MK-2870), a next-generation TROP2-directed antibody–drug conjugate with a novel topoisomerase I inhibitor payload, in combination with pembrolizumab and chemotherapy in the first-line setting, with objective response rate (ORR) and safety as primary endpoints (107). Other studies are exploring TROP2-targeting ADCs in later lines of treatment, including monotherapy approaches (e.g., NCT06356311 and NCT05489211) (108, 109).

Combination strategies are being increasingly explored not only in the metastatic setting but also in the perioperative setting, where the current standard of care is represented by perioperative chemotherapy with FLOT in combination with durvalumab, as established by the MATTERHORN trial (56). In this context, the ongoing phase II GEMINI perioperative study (NCT07069712) is evaluating novel immunotherapy-based combinations incorporating rilvegostomig, a PD-1/TIGIT bispecific antibody designed to provide dual immune checkpoint blockade within a single molecule and enhance antitumor T-cell responses. The aim of the study is to evaluate efficacy in terms of pathological complete response (pCR) and safety of different drug combinations across three distinct cohorts: patients with CLDN18.2-positive/HER2-negative tumors will receive sonesitatug vedotin (AZD0901), a CLDN18.2-targeted antibody–drug conjugate, in combination with rilvegostomig and fluoropyrimidine-based chemotherapy; patients with HER2-positive disease will receive trastuzumab deruxtecan in combination with rilvegostomig and chemotherapy; and patients with HER2-negative/CLDN18.2-negative tumors will receive rilvegostomig in combination with perioperative FLOT (110).

Beyond monoclonal antibody-based strategies, bispecific antibodies are emerging as a promising therapeutic approach in gastric cancer. In this context, givastomig, a Claudin 18.2/4-1BB bispecific antibody, has shown encouraging preliminary activity in early-phase clinical studies in pretreated patients when administered as monotherapy, with an objective response rate (ORR) of approximately 16% and a manageable safety profile, with no dose-limiting toxicities reported and a maximum tolerated dose not reached. Notably, givastomig is currently being explored in the first-line setting in combination with mFOLFOX6 and nivolumab in the ongoing phase I study NCT04900818 (111). Preliminary data from 17 patients treated with this combination have shown promising activity, with an ORR of 71% and a manageable safety profile, with the most common adverse events including nausea (47%), vomiting (29%), and infusion-related reactions (29%) (112).

### 4.2 Digital pathology, artificial intelligence, and multi-omics in poorly cohesive gastric carcinoma

Key unmet needs in PCGC include accurate detection of dispersed tumor cells and quantification of signet-ring cell components, as well as reliable biomarker-driven molecular and prognostic stratification. Digital pathology, multi-omics profiling, and artificial intelligence (AI)-based approaches may help address these challenges by enabling (i) diagnostic classification and subtyping, (ii) prognostic prediction, and (iii) treatment-response prediction.

Firstly, while recent classification systems have improved the pathological characterization of PCGC (2), translating these frameworks into consistently reproducible diagnoses remains challenging in routine practice. The diffuse growth pattern, marked cellular heterogeneity, and subtle morphological features characteristic of PCGC complicate conventional microscopic evaluation, particularly in limited biopsy samples and mixed-pattern tumors. As a result, inter-observer variability persists, impacting diagnostic accuracy and downstream clinical decision-making (1). In this context, the digitization of histological slides into highresolution whole-slide images and the use of AI have emerged as promising tools to address limitations of conventional microscopy, supporting more standardized and reproducible histopathological assessment (113–118).

Deep learning-based models trained on digitized WSIs have demonstrated strong performance in diagnostic tasks such as tumor detection and histological subtyping in GC, reducing inter-observer variability compared with conventional assessment (115, 119). Although most studies include GC broadly, emerging evidence suggests that AI models may be particularly effective in recognizing diffuse and poorly cohesive growth patterns, where morphological interpretation is most challenging (120, 121). In parallel, advances in radiomics and AI-based analysis of cross-sectional imaging have demonstrated the potential to capture quantitative features associated with tumor heterogeneity and molecular phenotype, with preliminary evidence suggesting improved discrimination of Lauren intestinal and diffuse histotypes and more refined patient stratification (122).

Beyond diagnosis, AI-enhanced digital pathology has extended toward prognostic modeling, where extracted histomorphological features are integrated into “pathomic” signatures associated with clinical outcomes (123, 124). Similarly, treatment-response prediction represents an emerging but more challenging application, in which deep learning models have been used to explore associations between histology and response to chemotherapy or immunotherapy in gastrointestinal cancers (125–127). AI approaches capable of automatically detecting key tumor cell types (128) and inferring molecular features such as microsatellite instability (MSI) status from hematoxylin and eosin slides (129) are under development and further highlight the potential of histology-based predictive modeling. Finally, multimodal AI frameworks integrating radiological imaging, whole-slide histopathology, and molecular data have shown promise for predicting treatment response and clinical outcomes in gastric cancer (130).

The integration of digital pathology with multi-omics data enables a linkage between histomorphological phenotypes and molecular drivers. This approach may be particularly relevant for molecularly defined subsets, including CDH1-mutant diffuse gastric cancers, where morphology, genotype, and clinical behavior are tightly linked (3). In parallel, spatially resolved multi-omics strategies have emerged as powerful approaches for dissecting tumor–stroma interactions and intratumoral heterogeneity (131, 132), two key determinants of the biological complexity and clinical behavior of PCGC (133). Finally, recent AI-based frameworks integrating spatial transcriptomics and histopathology further illustrate the potential of such multimodal approaches for dissecting tumor heterogeneity (134).

Significant challenges remain in the application of digital pathology and multi-omics technologies, integrated with AI-based approaches, in the oncological field. Overall, these limitations reflect both general difficulties inherent to AI-driven methodologies in oncology, including issues of data standardization, interpretability, and external validation (135), as well as PCGC-specific constraints such as the predominance of retrospective and small, highly selected cohorts, and the limited availability of robust, disease-specific datasets. Nevertheless, their convergence, coupled with large collaborative datasets, has the potential to transform the diagnosis, prognostic assessment, and therapeutic management of PCGC in the coming years.

## 5 Conclusions

Poorly cohesive gastric cancer is consistently associated with a worse prognosis compared with other histological subtypes and remains a clinically challenging entity. Despite the increasing availability of targeted therapies and immunotherapeutic approaches in gastric cancer, no treatment strategies are currently specifically tailored to this subgroup. Indeed, most available evidence supporting these therapies derives from biomarker-driven clinical trials, in which treatment benefit has been demonstrated at a molecular level but not specifically validated according to histological subtype. As a result, the clinical benefit of these approaches in poorly cohesive tumors remains incompletely defined, highlighting a critical unmet need.

In this evolving landscape, the role of the pathologist is becoming increasingly central, not only for accurate histological classification but also for the identification and validation of actionable biomarkers, which are essential to guide personalized therapeutic strategies. Importantly, the biological heterogeneity and unique microenvironmental features of poorly cohesive gastric cancer further emphasize the need for refined diagnostic and predictive tools.

In this context, the integration of digital pathology, artificial intelligence (AI), and multi-omics represents a promising and transformative approach to improve diagnostic reproducibility, enhance molecular stratification, and better capture tumor heterogeneity by linking histomorphology with underlying biological processes.

However, several challenges still limit the clinical implementation of these advances, including issues related to standardization, interpretability, regulatory validation, and integration into routine workflows, underscoring the continued need for expert pathological assessment.

Overall, bridging molecularly targeted therapies with advanced diagnostic technologies—while specifically addressing histology-driven gaps in current evidence—may pave the way toward a more precise and biologically informed management of poorly cohesive gastric cancer, ultimately aiming to improve patient outcomes in this particularly aggressive disease.
